# Phytosynthesis of Silver Nanoparticle (AgNPs) Using Aqueous Leaf Extract of *Knoxia sumatrensis* (Retz.) DC. and Their Multi-Potent Biological Activity: An Eco-Friendly Approach

**DOI:** 10.3390/molecules27227854

**Published:** 2022-11-14

**Authors:** Settu Loganathan, Kuppusamy Selvam, Muthugounder Subaramanian Shivakumar, Sengottayan Senthil-Nathan, Prabhakaran Vasantha-Srinivasan, Dhakshinamoorthy Gnana Prakash, Sengodan Karthi, Fahad Al-Misned, Shahid Mahboob, Ahmed Abdel-Megeed, Aml Ghaith, Patcharin Krutmuang

**Affiliations:** 1 Department of Botany, Periyar University, Salem 636011, Tamil Nadu, India; 2 Department of Anatomy, Saveetha Dental College and Hospital, Saveetha Institute of Medical and Technical Sciences, Chennai 600077, Tamil Nadu, India; 3 Department of Biotechnology, Periyar University, Periyar Palkalai Nagar, Salem 636011, Tamil Nadu, India; 4 Division of Biopesticides and Environmental Toxicology, Sri Paramakalyani Centre for Excellence and Environmental Sciences, Manonmaniam Sundaranar University, Alwarkurichi 627412, Tamil Nadu, India; 5 Department of Bioinformatics, Saveetha School of Engineering, Saveetha Institute of Medical and Technical Sciences (SIMATS), Chennai 602105, Tamil Nadu, India; 6 Department of Chemical Engineering, Sri Sivasubramaniya Nadar College of Engineering, Kalavakkam, Chennai 603110, Tamil Nadu, India; 7 Department of Entomology, University of Kentucky, Kentucky, KY 40503, USA; 8 Department of Zoology, College of Science, King Saud University, Riyadh 11451, Saudi Arabia; 9 Department of Plant Protection, Faculty of Agriculture Saba Basha Alexandria University, Alexandria 5452022, Egypt; 10 Department of Zoology, Faculty of Science, Derna University, Derna 20663, Libya; 11 Department of Entomology and Plant Pathology, Faculty of Agriculture, Chiang Mai University, Chiang Mai 50000, Thailand; 12 Innovative Agriculture Research Center, Faculty of Agriculture, Chiang Mai University, Chiang Mai 50000, Thailand

**Keywords:** silver nanoparticles, green synthesis, larvicide, anti-proliferation

## Abstract

Green synthesis of silver nanoparticles (AgNPs) has gained greater interest among chemists and researchers in this current scenario. The present research investigates the larvicidal and anti-proliferation activity of AgNPs derived from *Knoxia sumatrensis* aqueous leaf extract (*K. sumatrensis*-ALE) as a potential capping and reducing candidate. The synthesized AgNPs were characterized through-UV-spectra absorption peak at 425 nm. The XRD and FT-IR studied displayed the crystalline nature and presence of functional groups in prepared samples. FE-SEM showed the hexagonal shape of NPs with the size of 7.73 to 32.84 nm. The synthesized AgNPs displayed superior antioxidant and anti-proliferative activity (IC_50_ 53.29 µg/mL) of breast cancer cell line (MCF-7). Additionally, larvicidal activity against mosquito vector *Culex quinquefasciatus* larvae delivered (LC_50_-0.40, mg/L, and LC_90_-15.83) significant mortality rate post treatment with synthesized AgNPs. Overall, the present research illustrates that the synthesized AgNPs have high biological potential and present a perfect contender in the pharmacological and mosquitocidal arena.

## 1. Introduction

Noble metal nanoparticles (MNPs) have received considerable attention in recent years due to their various applications [1]. Various methods can achieve nanoparticle synthesis. Among these techniques, biological synthesis is rapid, simple and low cost and is the best method when compared to other synthesis methods [2]. The green synthesis process has benefits because it is an easy, inexpensive, and safe way to synthesize metal nanoparticles [3]. Additionally, employing plant extracts as a reducing agent makes it suitable for biomedical and pharmaceutical applications because no hazardous chemicals are utilized in the synthesis [4]. Recently, AgNPs have been synthesized using different plant species such as *Capsicum chinense* and *Hypericum perforatum* [5,6]. *Knoxia sumatrensis* belongs to the Rubiaceae. The plant has therapeutic uses in beverage preparation [7] and wound healing [8]. The extract of *K. sumatrensis* leaves was used to synthesize zinc oxide nanoparticles [9]. However, this is the first report on AgNPs synthesized by *K. sumatrensis* aqueous leaves extract.

Mosquitoes are considered to be global vectors of crucial diseases across the nation [10]. Dengue is spread by *Aedes aegypti* [11]. *Anopheles stephensi* is a carrier of plasmodium, which causes malaria [12]. *Culex quinquefasciatus* is a vector responsible for lymphatic filariasis [13]. The search for plant-based insecticides is underway, to find alternative mosquito control methods to chemical insecticides. Plant-based insecticides leave the least environmental footprint and can address the problem of insecticide resistance among mosquitoes to chemical insecticides [14]. Cancer is one of the leading diseases in developed countries. Its treatment primarily relies on surgery, chemo- and radio-therapies, although these are high cost, have side effects, and involve the death of normal cells along with cancer cells [15]. Consequently, novel chemotherapy drugs that have specific toxicity towards cancer cells and methods to directly target the tumor tissue are being actively explored. The use of nanoparticles with potential cytotoxic plant-based molecules is considered to be a vital way to treat cancer. Nanoparticles are efficient drug delivery vehicles that can be blended with bio-active plant compounds (secondary metabolites) derived from bio-rational plants for wide cancer therapeutic applications [16]. The present investigation highlights the characterization of green based Ag nanoparticles derived from white horseweed aqueous leaf extracts (*K. sumatrensis*-ALE) and their biological activity against the MCF-7 cell lines and larvicidal activity against the major mosquito vectors of medical importance.

## 2. Materials and Methods

### 2.1. Plant Collection and Extraction

*Knoxia sumatrensis* (Retz.) DC. was collected from Vytla hills Tamil Nadu, India, and authenticated by BSI. The leaves were washed, dried, and ground. Plant powder (10 g) was mixed with distilled water (100 mL) and boiled for 30 min at 70 °C. The *K. sumatrensis*-ALE was cooled and filtered with Whatman filter paper no.1 and stored at 4 °C for further use.

### 2.2. Synthesis

*K. sumatrensis*-ALE (10 mL) and 90 mL of silver nitrate (1 mM-AgNO_3_) were mixed and then stirred (3 h at 80 °C). After 3 h the color was changed from yellow into dark brown. The color indicated the formation of the AgNPs. The synthesized NPs were centrifuged at 10,000 for 15 min to obtain pellets, and then stored at 4 °C for further work.

### 2.3. Characterization

The synthesized AgNPs using *K. sumatrensis* extract were characterized by UV-Vis spectroscopy (UV-Vis-Shimadzu-1800). X-ray diffraction (XRD-Rigaku Miniflex) and analyzed by its crystalline structure. FT-IR indicated the functional groups, and nanoparticle morphology was analyzed by scanning electron microscopy (SEM- Jeol-6390LA).

### 2.4. Antioxidant

*K. sumatrensis* in synthesis of AgNPs was performed in DPPH by Shimada et al. [17], H_2_O_2_ Rajeshwar et al. [18], and ABTS assays by Giao et al. [19] with standard protocol and a dosage of 20–100 μg/mL. This assay used the standard of ascorbic acid. The assay inhibition (%) was calculated below.

(1) $$\mathrm{Scavenging}\;\mathrm{activity}\;=\left[\frac{\mathrm{Absorbance}\;\left(\mathrm{Control}\right)-\mathrm{Absorbance}\left(\mathrm{Sample}\right)}{\mathrm{Absorbance}\;\left(\mathrm{Control}\right)}\right]\times 100\%$$

### 2.5. Anti-Proliferative Activity

#### 2.5.1. Culture

Breast cancer cell-line (MCF-7) was obtained by NCCS-Pune, India, and it was grown in Dulbecco with DMEM elevated glucose medium.

#### 2.5.2. MTT Assay

Cell viability assays were described by Mosmann [20]. Ninety-six well plates were seeded in MCF-7 cells and maintained at 37 °C for 24 h in the incubator. The AgNPs dosages of 6.5, 12.5, 25, 50 and 100 µg/mL were used. Cell viability was calculated (MTT 10 µL for 4 h at 37 °C) after 24 h treatment. Dimethyl sulfoxide (DMSO) was dissolved in treated cells. The ELISA instrument (2.0-Epoch-USA) measured (OD-540 nm) the formazan in crystals (reference: 630 nm). Cell morphology was captured and calculated below.

Cell viability % = OD of AgNPs/OD of Control (Untreated) × 100%(2)

### 2.6. Larivicidal Activity

#### 2.6.1. Larval Culture

*Ae. aegypti*, *An. stephensi* and *Cx. quinquefasciatus* were collected from ICMR-VCRC Madurai) and maintained in the laboratory condition.

#### 2.6.2. Bioassay

This assay was followed by WHO [21] standard procedure with slight change (Thandapani et al. [22]). Larvae (4th instar) in twenty numbers were added from each cup (200 mL). The concentration of *K.sumatrensis*-ALE and synthesized AgNPs (5–25 mg/L) was added. The dead larva were counted after 12, 24 and 48 h post treatment then mortality (%) was obtained from average of *n* = 3. Abbott [23] was used in correction of larval mortality.

### 2.7. Statistical Analysis

The data were analyzed by mean ± SD. Larval mortality was calculated by Probit analysis for finding out LC_50_, LC_90_ and chi-square values using SPSS software. All the data were analyzed with analysis of variance (ANOVA), and treatment means were compared by Tukey’s family error test (*p* < 0.05) for pairwise comparison using Minitab^®^ 16 software package.

## 3. Results and Discussion

### 3.1. UV-Vis Spectral Analysis

**Figure 1:**
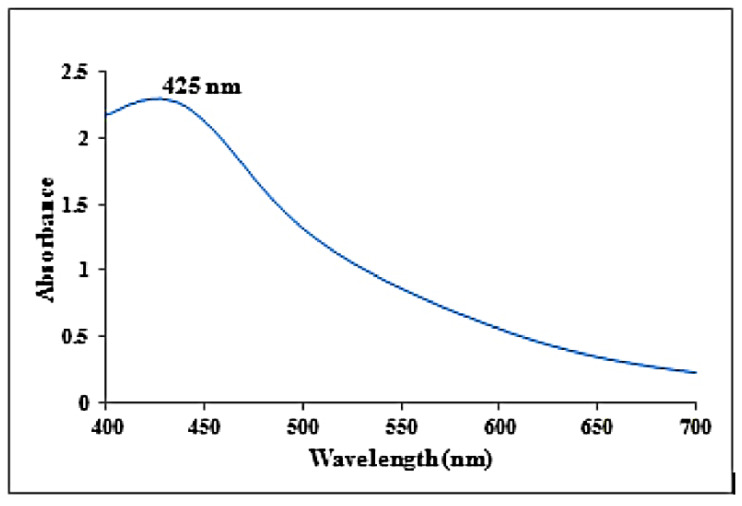
UV-visible absorbance spectra obtained from synthesized silver nanoparticles using *K. sumatrensis* aqueous leaf extracts (*Ks*-ALE).

The synthesized AgNPs from *K.sumatrensis* extract showed the UV absorption peak at 425 nm due to the surface plasmon resonance (SPR), as shown in Figure 1. The mechanism of green synthesis of silver nanoparticles is due the constituent secondary metabolites donating electrons for the reduction of Ag+ ions to Ag° ions [24]. As compared to our results, the recent reports of silver nanoparticles synthesized from *Passiflora subpeltata* leaf extract demonstrated the UV absorption peak at 456 nm [16]. Additionally, the AgNPs of *Acacia concinna* leaf extract exhibited the UV absorption peak at 440 nm [25].

### 3.2. X-ray Diffraction Studies

**Figure 2:**
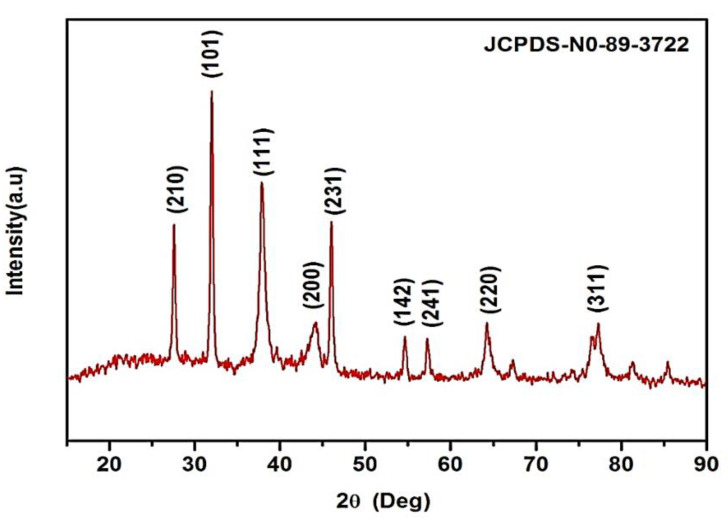
XRD pattern of synthesized AgNPs using *K. sumatrensis* aqueous leaf extracts (*Ks*-ALE) exhibiting the facets of crystalline silver.

*K. sumatrensis* of synthesized AgNPs in XRD results showed the nine peaks at 2θ degree ranges of 27.87°, 32.14°, 38.77°, 44.20°, 46.10°, 54.40°, 57.34°, 64.34° and 77.72° corresponding to values 210, 101, 111, 200, 231, 142, 241, 220 and 311 (Figure 2). The lattice planes of pure silver based on the face-center cubic structure (JCPDS No. 89–3722) and the XRD results indicate the crystalline nature of AgNPs. Similar data were found for synthesized AgNPs using *Sargassum myriocystum* extract [26]. In another report, *Drimia polyantha* derived AgNPs revealed the presence of four diffraction values at 2θ ranges [27]. The average crystal size of synthesized AgNPs was 15.70 nm using Debye Scherrer’s formula.

### 3.3. FT-IR Study

**Figure 3:**
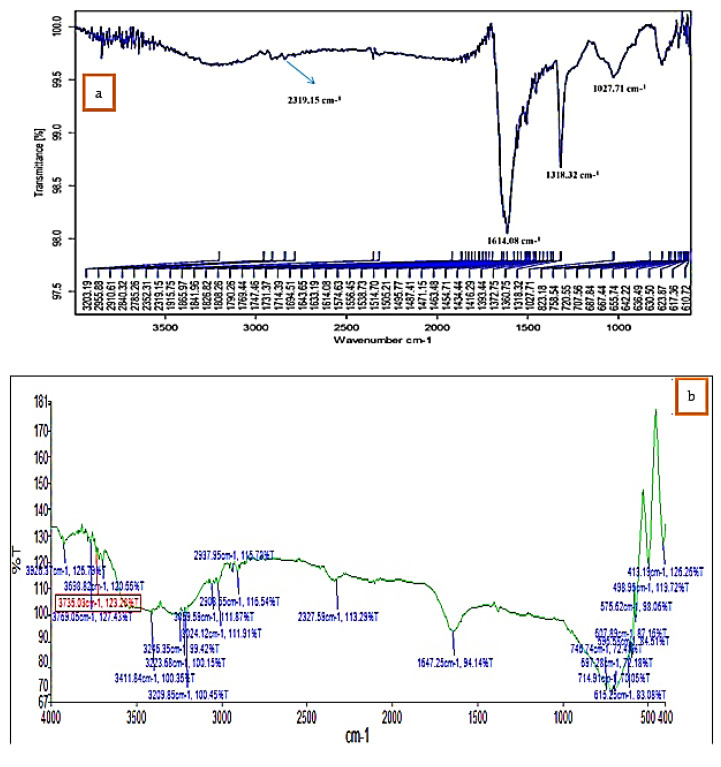
FT−IR analysis of (**a**) *K. sumatrensis* aqueous leaf extracts (*Ks*-ALE). (**b**) Synthesized AgNPs.

FTIR analysis confirmed that the bioproduction of Ag^+^ ions to silver nanoparticles was due to the reduction of capping material of green extract. The IR spectra of *K. sumatrensis* aqueous leaf extract revealed four functional groups: 3411.84 cm^−1^ for OH (alcohols, phenols), 3209.85 cm^−1^ for OH (carboxylic acids), 2908.55 cm^−1^ for C-H (alkanes), 2327.59 cm^−1^ for P-H (phosphines) and 1647.25 cm^−1^ for C=C (alkene) (Figure 3; Table 1). The synthesized AgNPs of *K.sumatrensis* extract in IR spectra showed the four functional groups as: 2319 cm^−1^ for CH (methylene), 1614.08 cm^−1^ by C=C stretching (alkenes), 1318.32 cm^−1^ indicating C-N (amines) and 1027.71 cm^−1^ C-X stretching (fluoride group) (Figure 3b; Table 2. Kumar et al. [28] reported that the carboxyl (–C=O), hydroxyl (–OH) and amine (–NH) groups of leaf extracts are importantly involved in fabrication of silver nanoparticles. Previous research of Morales-Lozoya et al. [29] illustrates that the synthesized AgNPs from *Moringa citrifolia* extract showed the presence of four functional groups. Correspondingly, the synthesized AgNPs by *Cucumis prophetarum* extract indicated the existence of eight functional groups [30]. The essential functional groups, such as alcohol, amides, alkanes, methyl, aliphatic and halides, confirmed the presence of NPs. They were stabilizing, capping and dipping agents of the AgNPs [31]. Thus, in this study, *K. Sumatrensis* derived AgNPs functional groups may be responsible for the formation of AgNPs.

### 3.4. SEM and EDAX Analysis

**Figure 4:**
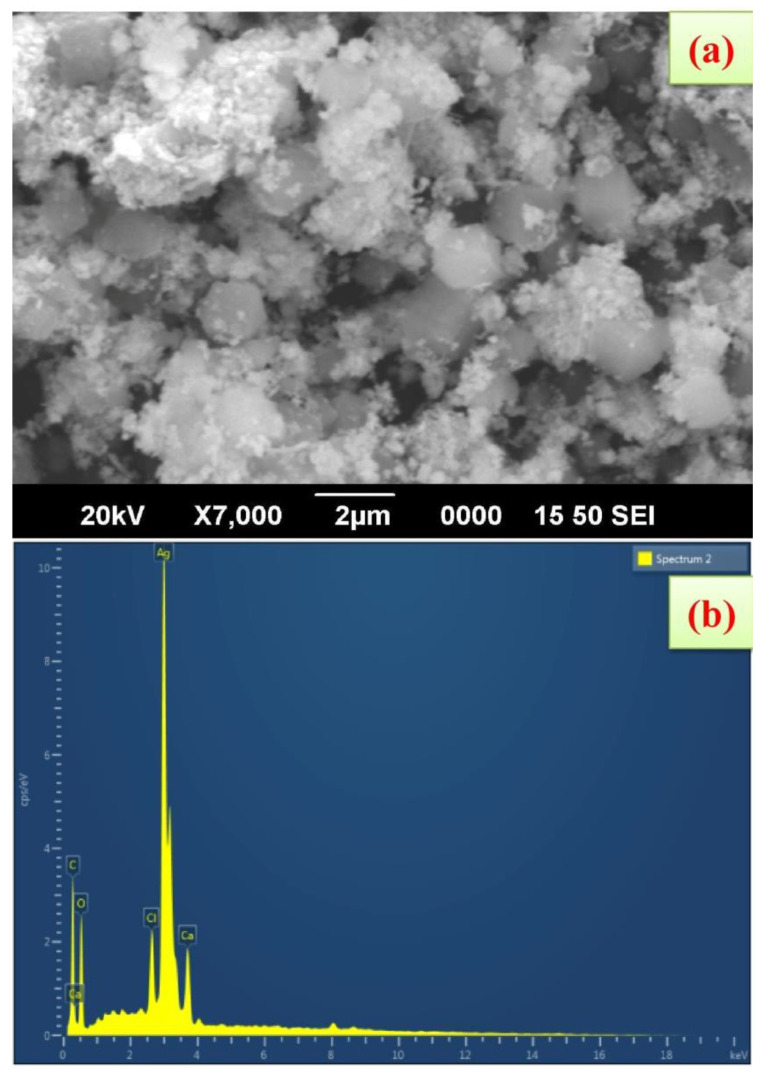
Scanning electron microscope images of AgNPs synthesized using *K. sumatrensis* aqueous leaf extracts (*Ks*-ALE); (**a**) 2 µm; (**b**) EDAX spectrum analysis confirmed the presence of silver signals.

FE-SEM shows that the synthesized AgNPs using *K. sumartrensis* extract had hexagonal shapes (Figure 4). In parallel, the related shape was obtained by *Calotropis gigantean* and *Sargassum myriocystum* in synthesized AgNPs [25,32]. In the present study, the chemical composition was analyzed with EDX, and silver, carbon, oxygen, calcium and chlorine were present (Figure 4). Our research was in agreement with the previous investigation, which found that synthesized AgNPs of *Plumeria alba* leaf extract in EDX confirmed the presence of silver [33].

### 3.5. Antioxidant Activity

*K. sumatrensis* of synthesized AgNPs proved to have superior antioxidant activity against DPPH, ABTS and hydroxyl radical assays. In all three assays, inhibition percentages in synthesized AgNPs (55.20%, 53.15 and 53.70) and ascorbic acid (65.30%, 62.20 and 68.45), respectively, at higher concentrations of 100 µg/mL, were seen (Supplementary Material Figures S1–S3) in dose-dependent manner. Correspondingly, synthesized AgNPs derived from *Eucalyptus tereticornis,* commonly known as forest red gum, and *Embeliaribes* (common name: false black pepper), showed potential antioxidant activity [34,35]. The synthesized AgNPs and ascorbic acid (standard) DPPH IC_50_ values were calculated to be 92.73 and 79.37, respectively. In parallel, the synthesized AgNPs derived from *Nepeta leucophylla* (common name: white leaved catmint) root extract delivered significant antioxidant activity (IC_50_ value of 119.28 µg/mL) [36]. Previous research illustrates that the DPPH radical scavenging capacity of the synthesized AgNPs showed the presence of phenolic derivatives that can deliver the H into OH groups [37]. The ABTS inhibitory concentration (IC_50_) of the synthesized AgNPs displayed 94.68 μg/mL as compared to standard IC_50_ value of 79.48 µg/mL, and it is in parallel with the previous investigation on *Cymbopogon citrates* (common name: lemon grass) derived AgNPs, which revealed the IC_50_ value of 123.89 µg/mL [38]. The H_2_O_2_-IC_50_ value of the synthesized AgNPs showed significant range of 91.46 μg/mL as compared to the standard (IC_50_:71.47 μg/mL). The present research was well matched with the previous findings that *Cassia angustifolia* (common name: Indian senn) flowers extract of AgNPs delivered IC_50_ value of 78.10 µg/mL [39].

### 3.6. Anti-Proliferative Activity

Figures S4 and S5 show the cell viability (%) and treated images of MCF-7 cell line by the synthesized AgNPs from *K. sumatrensis* extract in a dose dependent manner. The percentage of viability is directly proportional to the synthesized AgNPs dosages (6.5 to 100 µg/mL) treatments. The inhibitory dosage IC_50_ of synthesized *K. sumatrensis* was 5.3 at 29 µg/mL at the maximum dosage of 100 µg/mL. Correspondingly, the synthesized AgNPs from *Syzgium aromaticum* (common name: clove) delivered profound MCF-7 activity (IC_50_-60 µg/mL) [40]. Similar results were obtained in the treatment of *Camellia sinensis* (common name: tea shrub) (IC_50_-59.2 µg/mL) [41] and also in *Cassia angustifolia* (Indian senna) (IC_50_-73.82 µg/mL) [39]. All the above findings were in line with our present investigation, which reveals that the *K. sumatrensis* of AgNPs delivered significant MCF-7 activity.

### 3.7. Larvicidal Activity

*K. sumatrensis*-ALE and the synthesized AgNPs displayed significant larvicidal activity against all three crucial mosquito vectors and the mortality rate was significant in the filarial vector *Cx. quinquefasciatus* as compared to dengue (Ae. aegypti) and malarial vector (*A. stephensi*) in the preliminary screening. The lethal concentrations (LC_50_ and LC_90_) were displayed at 0.40 mg/L and 15.83 mg/L, respectively, post 48 h treatment with *K. sumatrensis*-ALE (Tables S1–S3). In parallel, the synthesized AgNPs derived from *Ixorabrachiata* (Gorbale) and *Carmona retusa* (Fukien tea tree) extract delivered significant larvicidal activity against the filarial vector *Cx. quinquefasciatus* [42,43].

## 4. Conclusions

As an endnote, the present biological screening of green synthesized *K. sumatrensis*-ALE displayed multipotent biological activity as an anti-cancer and larvicidal agent and the present baseline toxicological screening suggests further interest in this eco-friendly green extract derived from white horseweed, as a potential eco-friendly drug and mosquitocide. Future prospective research is highly required to determine the molecular mechanisms and pharmacokinetic activity of bioactive molecules derived from *K. sumatrensis* using an in vivo rat model and field trial to determine the mosquitocidal actions and their non-target toxicity against beneficial insects which share the same ecological niche of mosquito vectors.

## Figures and Tables

**Table 1:** FT-IR analysis of *K. sumatrensis* aqueous leaf extract (*Ks*-ALE).

Wave Number (CM^−1^)	Intensity	Group Compound	Functional Group
3411.84	Medium	OH	Alcohols, Phenols
3209.85	Medium	OH	Carboxylic acids
2908.55	Strong	C-H	Alkanes
2327.59	Medium	P-H	Phosphines
1647.25	Medium-Strong	C=C	Alkene

**Table 2:** FT-IR analysis of AgNPs using *K. sumatrensis* aqueous leaf extract (*Ks*-ALE).

Wave Number (CM^−1^)	Intensity	Group Compound	Functional Group
2319.15	Strong	CH	Methylene
1614.08	Medium	C=C	Alkenes
1318.32	Medium-Strong	C–N	Amines
1027.71	Strong	C–X	Fluoride

## Data Availability

All relevant data are within the paper and its Supporting Information files.

## Supplementary Materials

The following supporting information can be downloaded at: https://www.mdpi.com/article/10.3390/molecules27227854/s1. Figure S1: Antioxidant activity of AgNPs synthesized using *K. sumatrensis* aqueous leaf extracts (*Ks*-ALE). DPPH radical scavenging activity, (b) ABTS radical scavenging activity and (c) Hydroxyl scavenging activity. Figure S4: MTT assay confirming the Anti-proliferative effects of AgNPs using aqueous leaf extracts of *K. sumatrensis* (*Ks*-ALE) against MCF-7 cell line. Figure S5: Anti-proliferative observed from confocal microscope (340 pixel); Control and various concentrations (6.5, 12.5, 25, 50 and 100 µg/mL) of *K. sumatrensis* aqueous leaf extract (*Ks*-ALE) of AgNPstreated on breast cancer cells (MCF-7). Table S1: Larvicidal activity of *K. sumatrensis* aqueous leaf extract (*Ks*-ALE) and Synthesized AgNPs against *Aedes aegypti.* TableS2: Larvicidal activity of *K. sumatensis* aqueous leaf extract (*Ks*-ALE) and synthesized AgNPs against *Anopheles stephensi.* Table S3: Larvicidal activity of *K. sumatrensis* aqueous leaf extract (*Ks*-ALE) and synthesized AgNPs against *Culex quinquefasciatus.*

## Sample Availability

Samples of the compounds Silver Nanoparticle (AgNPs) of *Knoxia Sumatrensis* are currently not available from the authors.
