# Supplementary material for: Phytosynthesis of Silver Nanoparticle (AgNPs) Using Aqueous Leaf Extract of Knoxia sumatrensis (Retz.) DC. and Their Multi-Potent Biological Activity: An Eco-Friendly Approach
Source: Molecules. 2022 Nov 14;27(22):7854. doi: 10.3390/molecules27227854 (PMC9694222; doi:10.3390/molecules27227854)
Supplement: Supplementary file 1 [file molecules-27-07854-s001.zip › molecules-1954235-supplementary.pdf]

Supplementary Materials

Phytosynthesis of Silver Nanoparticle (AgNPs) Using Aqueous Leaf Extract of *Knoxia Sumatrensis* (Retz.) DC. and Their Multi-Potent Biological Activity: An Eco-Friendly Approach

Settu Loganathan [1,2], Kuppusamy Selvam [1], Muthugounder Subaramanian Shivakumar [3], Sengottayan Senthil-Nathan [4,*], Prabhakaran Vasantha-Srinivasan [5], Dhakshinamoorthy Gnana Prakash [6], Sengodan Karthi [7], Fahad Al-Misned [8], Shahid Mahboob [8], Ahmed Abdel-Megeed [9], Aml Ghaith [10] and Patcharin Krutmuang [11,12,*]

1 Department of Botany, Periyar University, Salem 636011, Tamil Nadu, India
2 Department of Anatomy, Saveetha Dental College and Hospital, Saveetha Institute of Medical and Technical Sciences, Chennai 600077, Tamil Nadu, India
3 Department of Biotechnology, Periyar University, Periyar Palkalai Nagar, Salem 636011, Tamil Nadu, India
4 Division of Biopesticides and Environmental Toxicology, Sri Paramakalyani Centre for Excellence and Environmental Sciences, Manonmaniam Sundaranar University, Alwarkurichi 627412, Tamil Nadu, India
5 Department of Bioinformatics, Saveetha School of Engineering, Saveetha Institute of Medical and Technical Sciences (SIMATS), Chennai 602105, Tamil Nadu, India
6 Department of Chemical Engineering, Sri Sivasubramaniya Nadar College of Engineering, Kalavakkam, Chennai 603110, Tamil Nadu, India
7 Department of Entomology, University of Kentucky, Kentucky, KY 40503, USA
8 Department of Zoology, College of Science, King Saud University, Riyadh 11451, Saudi Arabia
9 Department of Plant Protection, Faculty of Agriculture Saba Basha Alexandria University, Alexandria 5452022, Egypt
10 Department of Zoology, Faculty of Science, Derna University, Derna, Libya
11 Department of Entomology and Plant Pathology, Faculty of Agriculture, Chiang Mai University, Chiang Mai 50000, Thailand
12 Innovative Agriculture Research Center, Faculty of Agriculture, Chiang Mai University, Chiang Mai 50000, Thailand
* Correspondence: senthil@msuniv.ac.in (S.S.-N.); patcharink26@gmail.com (P.K.)

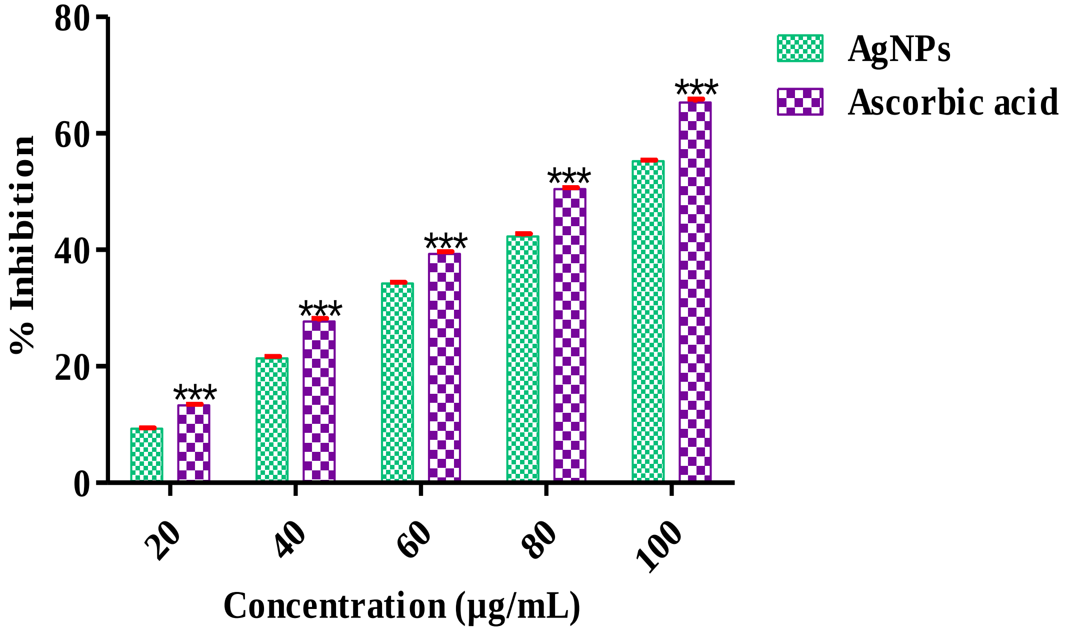

Figure S1. Antioxidant activity of AgNPs synthesized using *K. sumatrensis* aqueous leaf extracts (*Ks*-ALE). (**a**) DPPH radical scavenging activity. The values are expressed as mean ± SD values and analyzed by Two-Way analysis of variance (ANOVA). Asterisk (***) indicates significant different among treatments with respect to control ($p < 0.001$).

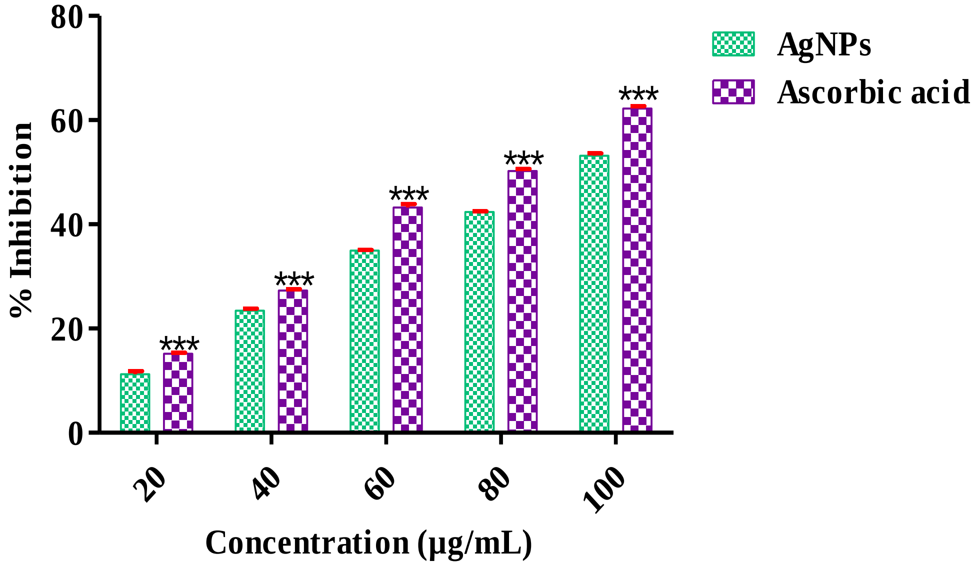

Figure S2. Antioxidant activity of AgNPs synthesized using *K. sumatrensis* aqueous leaf extracts (*Ks*-ALE) (**b**) ABTS radical scavenging activity. The values are expressed as mean ± SD values and analyzed by Two- Way analysis of variance (ANOVA). Asterisk (***) indicates significant different among treatments with respect to control ($p < 0.001$).

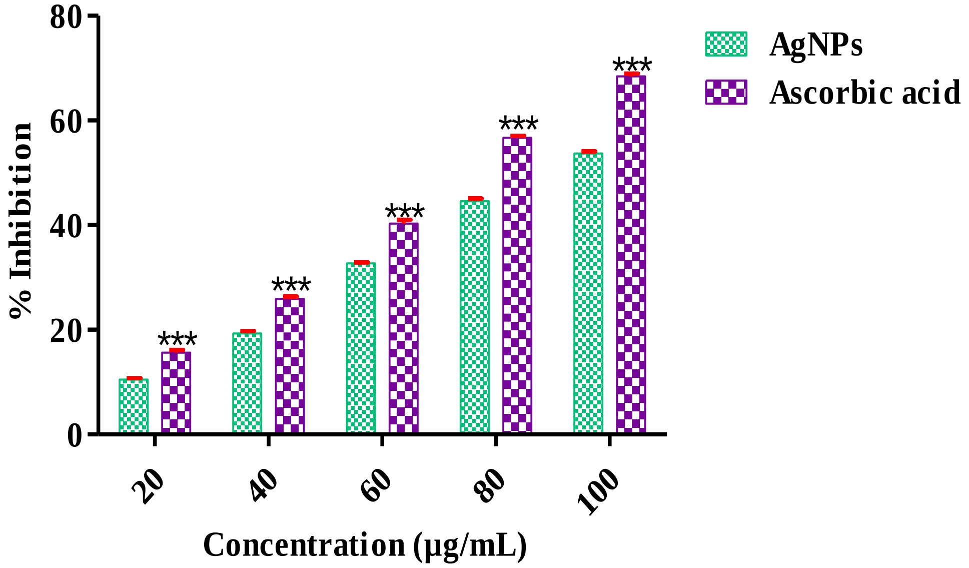

Figure S3. Antioxidant activity of AgNPs synthesized using *K. sumatrensis* aqueous leaf extracts (*Ks*-ALE) (**c**) Hydroxyl scavenging activity. The values are expressed as mean ± SD values and analyzed by Two-Way analysis of variance (ANOVA). Asterisk (***) indicates significant different among treatments with respect to control ($p < 0.001$).

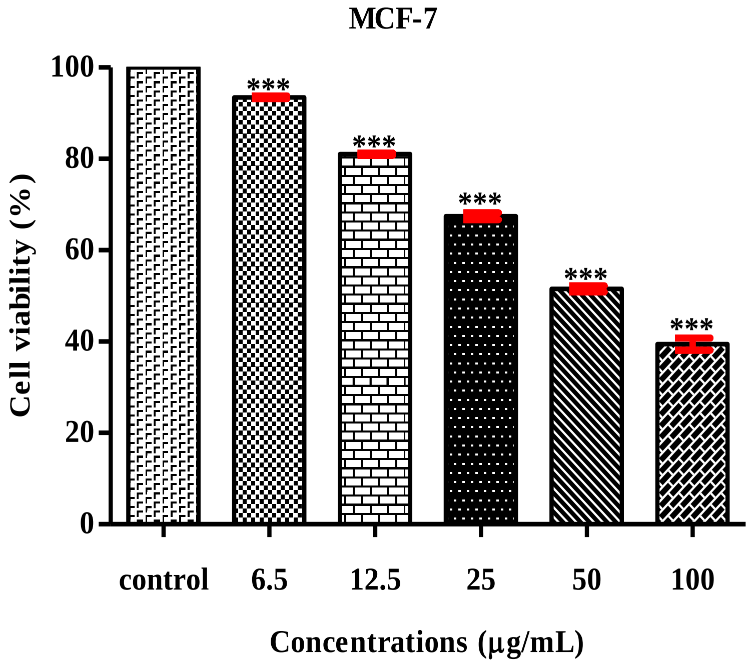

Figure S4. MTT assay confirming the Anti-proliferative effects of AgNPs using aqueous leaf extracts of *K. sumatrensis* (*Ks*-ALE) against MCF-7 cell line. The values are expressed as mean ± SD values and analyzed by One - way analysis of variance (ANOVA). Asterisk (***) indicates significant different among treatments with respect to control($p < 0.001$).

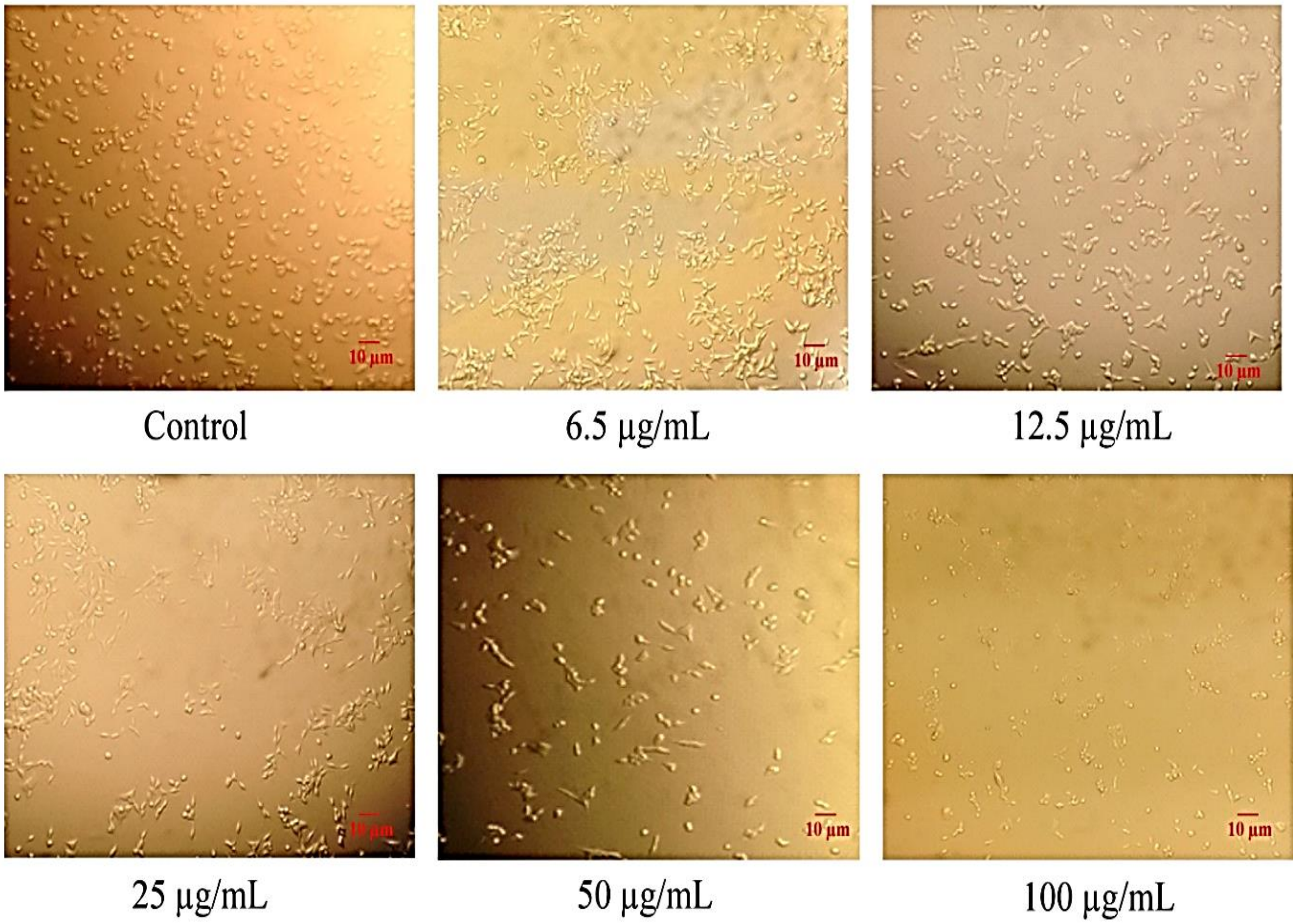

Figure S5. Anti-proliferative observed from confocal microscope (340 pixel); Control and various concentrations (6.5, 12.5, 25, 50 and 100 µg/mL) of *K. sumatrensis* aqueous leaf extract (*Ks*-ALE) of AgNPs treated on breast cancer cells (MCF-7).

Table S1. Larvicidal activity of *K. sumatrensis* aqueous leaf extract (*Ks*-ALE) and Synthesized AgNPs against *Aedes aegypti.*

Time (Hour)	Samples	LC_{50} (mg/mL) (LCL-UCL)	LC_{90} (mg/mL) (LCL-UCL)	χ^2	*Df*
12	Plant extract	37.36 (29.08–66.62)	67.29 (48.42–137.29)	1.79	13
	AgNPs	21.81 (18.15–29.49)	51.72 (39.47–86.84)	1.58	13
24	Plant extract	26.01 (21.88–35.34)	52.35 (40.70–82.84)	1.32	13
	AgNPs	9.56 (6.31–11.82)	27.70 (23.92–34.52)	1.09	13
48	Plant extract	16.06 (13.05–19.47)	41.56 (33.42–60.71)	0.76	13
	AgNPs	4.46 (3.30–5.42)	17.62 (15.21–21.54)	4.46	13

LC_{50}: Lethal concentration kills 50% of the exposed larvae, LC_{90}: Lethal concentration kills 90% of the exposed larvae, LCL: Lower confidence limit, UCL: Upper confidence limit, χ^2 Chi-square value, *df*; degrees of freedom.

Table S2. Larvicidal activity of *K. sumatensis* aqueous leaf extract (*Ks*-ALE) and synthesized AgNPs against *Anopheles stephensi.*

Time (Hour)	Samples	LC_{50} (mg/mL) (LCL-UCL)	LC_{90} (mg/mL) (LCL-UCL)	χ^2	*Df*
12	Plant extract	28.13 (23.92–37.23)	50.87 (40.47–75.78)	1.38	13
	AgNPs	16.86 (13.76–20.77)	43.76 (34.70–66.30)	1.04	13
24	Plant extract	24.24 (20.34–32.92)	30.75 (26.78–37.61)	0.867	13
	AgNPs	5.04 (7.73–11.36)	24.30 (20.84–31.14)	2.15	13
48	Plant extract	15.54 (12.32–18.99)	42.05 (33.54–62.83)	0.812	13
	AgNPs	3.68 (0.99–7.70)	18.94 (16.47–23.00)	3.94	13

LC_{50}: Lethal concentration kills 50% of the exposed larvae, LC_{90}: Lethal concentration kills 90% of the exposed larvae, LCL: Lower confidence limit, UCL: Upper confidence limit, χ^2 Chi-square value, *df*; degrees of freedom.

Table S3. Larvicidal activity of *K. sumatrensis* aqueous leaf extract (*Ks*-ALE) and synthesized AgNPs against *Culex quinquefasciatus.*

Time (Hours)	Samples	LC_{50} (mg/mL) (LCL-UCL)	LC_{90}(mg/mL) (LCL-UCL)	χ^2	*Df*
12	Plant extract	25.06 (21.45–32.37)	48.85 (38.97–72.17)	1.19	13
	AgNPs	11.25 (8.47–13.38)	29.28 (25.29–36.44)	1.65	13
24	Plant extract	20.68 (17.33–26.92)	49.06 (38.03–78.35)	0.93	13
	AgNPs	2.60 (3.24–5.81)	19.30 (16.65–23.78)	3.91	13
48	Plant extract	10.99 (6.99–13.72)	34.60 (28.52–47.88)	0.85	13
	AgNPs	0.40 (6.64–3.98)	15.83 (13.48–19.51)	2.62	13

LC_{50}: Lethal concentration kills 50% of the exposed larvae, LC_{90}: Lethal concentration kills 90% of the exposed larvae, LCL: Lower confidence limit, UCL: Upper confidence limit, χ^2 Chi-square value, *df*; degrees of freedom.
